# Efficacy of customized corneal crosslinking versus standard corneal crosslinking in patients with progressive keratoconus (C-CROSS study): study protocol for a randomized controlled trial

**DOI:** 10.1186/s12886-023-02976-4

**Published:** 2023-05-19

**Authors:** Magali M. S. Vandevenne, Tos T. J. M. Berendschot, Bjorn Winkens, Frank J. H. M. van den Biggelaar, Nienke Visser, Mor M. Dickman, Robert P. L. Wisse, Robert-Jan H. J. Wijdh, Abhijit Sinha Roy, Rohit Shetty, Rudy M. M. A. Nuijts

**Affiliations:** 1 University Eye Clinic Maastricht, Maastricht University Medical Centre+, P Debyelaan 25, Maastricht, 6229 HX the Netherlands; 2 School for Mental Health and Neuroscience (MHeNS), Maastricht University, Maastricht, the Netherlands; 3 Department of Methodology and Statistics, Faculty of Health, Medicine, and Life Sciences (FHML), Care and Public Health Research Institute (CAPHRI), Maastricht University, Maastricht, the Netherlands; 4 Ophthalmology Department, University Medical Centre Utrecht, Utrecht, the Netherlands; 5 Ophthalmology Department, University Medical Centre Groningen, Groningen, the Netherlands; 6 Narayana Nethralaya Eye Hospital, Bangalore, India

**Keywords:** Keratoconus, Crosslinking, Customized

## Abstract

**Background:**

Keratoconus is a degenerative disorder of the cornea leading to a protrusion and thinning with loss of visual acuity. The only treatment to halt the progression is corneal crosslinking (CXL), which uses riboflavin and UV-A light to stiffen the cornea. Recent ultra-structural examinations show that the disease is regional and does not affect the entire cornea. Treating only the affected zone with CXL could be as good as the standard CXL, that treats the entire cornea.

**Methods:**

We set up a multicentre non-inferiority randomized controlled clinical trial comparing standard CXL (sCXL) and customized CXL (cCXL). Patients between 16 and 45 years old with progressive keratoconus were included. Progression is based on one or more of the following changes within 12 months: 1 dioptre (D) increase in keratometry (Kmax, K1, K2); or 10% decrease of corneal thickness; or 1 D increase in myopia or refractive astigmatism, requiring corneal crosslinking.

**Discussion:**

The goal of this study is to evaluate whether the effectiveness of cCXL is non-inferior to sCXL in terms of flattening of the cornea and halting keratoconus progression. Treating only the affected zone could be beneficial for minimalizing the risk of damaging surrounding tissues and faster wound healing. Recent non-randomized studies suggest that a customized crosslinking protocol based on the tomography of the patient’s cornea may stop the progression of keratoconus and result in flattening of the cornea.

**Trial registration:**

This study was prospectively registered at ClinicalTrials.gov on August 31^st^, 2020, the identifier of the study is NCT04532788.

## Background

Keratoconus is a degenerative disorder of the cornea involving disruption and loss of the native collagen network leading to severe corneal thinning [1–3], which eventually results in a typical cone shaped cornea causing irregular astigmatism and impaired visual acuity [4]. Recent ultra-structural examinations show that the disease does not affect the entire cornea in its early stage, but rather starts locally [1, 5].

The onset of keratoconus is during puberty and gradually progresses until the mid-20 s and 30 s [4]. Around the age of 40 the disease stabilizes, showing hardly any progression in patients older than 45. With a prevalence of approximately 265 cases per 100,000 individuals and an incidence of approximately 13 cases per 100,000 in the Netherlands, the disorder is relatively common [6]. While the cause of keratoconus is not fully understood, disease progression seems to be influenced by genetic and environmental factors. An increased prevalence of the disease has been associated with systemic disorders e.g., atopy, Down syndrome, and Marfan syndrome [7].

The treatment for keratoconus depends on the severity of the disease [8]. In its initial stage, treatment aims at improving visual acuity. This can be achieved with glasses and specialized contact lenses but also by implanting intrastromal corneal ring segments to regularize the corneal shape. Although these treatments may improve visual acuity, they do not cure keratoconus.

Until two decades ago the only treatment for advanced keratoconus was corneal transplantation, an invasive technique with a chance of corneal graft rejection and the need of retreatment after a few years [9, 10]. In addition, corneal transplantation has an important impact on the quality of life and the outcome may be suboptimal because of significant post-keratoplasty astigmatism.

In 2003 Wollensak et al. introduced corneal crosslinking (CXL) to halt the progression of keratoconus in humans [11]. First the top layer of the cornea, the epithelium, is debrided after which the cornea is soaked for 30 min with the photosensitizer riboflavin. Hereafter a 9.0 mm diameter Ultraviolet-A (UV-A) beam radiates the cornea for 30 min with a fluence of 3 mW/cm^2^ resulting in a total energy of 5.4 J/cm^2^. This protocol is called the Dresden protocol. Currently, accelerated versions of the Dresden protocol are used in clinical practice with different fluences of 9mW/cm^2^, 10mW/cm^2^ and 15 mW/cm^2^. The higher the fluence, the shorter the treatment time, in which, according to the Bunsen–Roscoe reciprocity law, the total amount of energy stays the same [12–14]. During the corneal crosslinking oxygen radicals are formed that interact with the surrounding molecules, leading to the formation of new chemical bounds between the collagen fibrils (i.e. corneal crosslinks) [15], with the final goal to stiffen the cornea and halt the progression of the disease.

For any treatment, it is desirable that unaffected regions of the tissue involved are not unnecessarily treated by an intervention or drug application. To minimize the risk of damage to surrounding tissues in CXL it would be beneficial that the UVA beam is restricted only to the affected, keratoconic zone in the patient’s cornea [16]. This can be achieved by *customizing* the beam shape and size in a way that only the degenerated zone is treated, i.e., by customized crosslinking (*c*CXL). Recently, studies provided clinical evidence that similar clinical outcomes can be achieved if only the cone is treated rather than the whole cornea [17–20]. We hypothesized based on the results of these previous studies that only treating the affected zone has three potential benefits: (1) a faster recovery (e.g. increased corneal reepithelialisation), (2) stronger flattening of maximum keratometry and (3) better visual outcome (corrected distance visual acuity, CDVA). However, since none of these studies were randomized and study results were limited by small sample sizes, there is a need for a randomized controlled trial with an appropriate design and sample size to confirm these findings. This study will be setup as such, with the aim to investigate whether *c*CXL is non-inferior to *s*CXL in terms of halting the disease progression and flattening of the corneal surface.

## Methods

### Objectives

The primary objective is the change in maximum keratometry (Kmax) measured with a Scheimpflug-based tomographer (Pentacam® HR, OCULUS Optikgeraete GmbH, Wetzlar, Germany). Under the subheading ‘Outcome measures’ all the secondary objectives are summed up and explained.

### Study design

We will set up a multicentre non-inferiority randomized controlled clinical trial at Maastricht University Medical Centre + , University Medical Centre Utrecht and University Medical Centre Groningen.

### Study population

Patients will be included from the outpatient ophthalmology department at the three academic centres. Inclusion criteria are patients between the age of 16 and 45 years old with progressive keratoconus based on one or more of the following changes within 12 months: 1 dioptre (D) increase in keratometry (Kmax, K1, K2) or 10% decrease of corneal thickness or 1 D increase in myopia or refractive astigmatism. Exclusion criteria are corneal scarring, corneal diseases other than keratoconus, history of corneal surgery (e.g. refractive surgery, corneal transplantation, intracorneal ring segments), unwilling or unable to give informed consent, unwilling to accept randomization or inability to complete follow-up (e.g. hospital visits) or comply with study procedures, insufficient corneal thickness including epithelium < 400 µm, pregnancy, if both eyes are eligible only the first eye which is undergoing corneal crosslinking is enrolled in the study, or participation in another clinical study.

### Study procedures

#### Crosslinking procedures

**Fig. 1:**
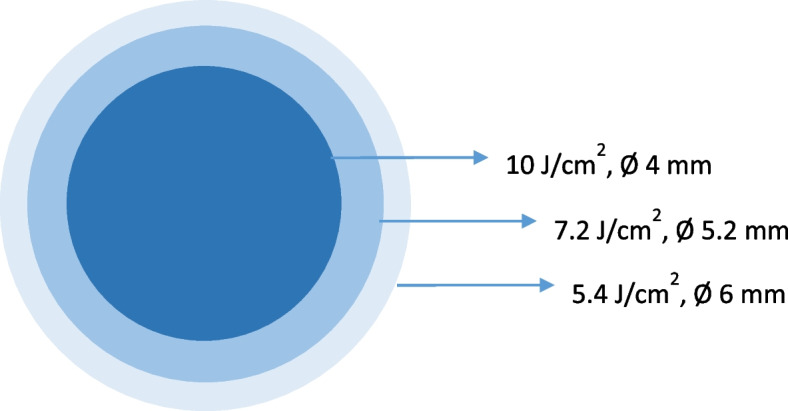
Customized treatment pattern

Before the CXL procedure the treatment location of the customized procedure needs to be determined. A patient-specific treatment pattern, based on the patient’s Pentacam images, will be used to treat the cornea. The CXL treatment pattern exists out of three circles and is centred on the cone. To estimate the cone location a combination of the thinnest corneal point, maximum anterior elevation and maximum posterior elevation is used. The average of these three points is calculated and functions as the centre for the treatment pattern. The treatment pattern itself consists of three concentric circles. The diameter of the smallest circle is 4 mm, of the middle circle 5.2 mm and of the biggest circle is 6 mm. Each circle receives a different amount of energy, which gradually decreases with increasing circle size. The smallest circle receives the highest amount of energy, 10 J/cm^2^, this is equal to a fluence of 10 mW/cm^2^ for 16.7 min. The middle circle receives 7.2 J/cm^2^ and the biggest circles receives 5.4 J/cm^2^ (Fig. 1). The patient-specific treatment pattern will be marked on the cornea centred on the cone location.

The first step of the CXL procedure is the removal of the epithelium. In the sCXL protocol, the epithelium is debrided with alcohol 20% over a region with a diameter of 9.0 mm. In the cCXL the cone location is marked and the epithelium is removed with alcohol 20% over a region with a diameter of 6.0 mm. After the removal of the epithelium the pachymetry is measured, if it is more than 400 µm, the cornea is soaked with 0.1% riboflavin, saline, HPMC (VibeX RapidTM) 1 drop every 2 min for 10 min. The pachymetry is measured a second time. If the pachymetry is less than 400 µm, hypotonic riboflavin is applied until the cornea is thicker than 400 µm. After the application of riboflavin, the cornea is irradiated with UV-A light. In sCXL a fluence of 10 mW/cm^2^ is used during 9 min with a diameter of 9.0 mm, this gives a total energy of 5.4 J/cm^2^. In cCXL the cornea is irradiated with UVA-light according to the treatment pattern mentioned earlier. Both procedures are done with the Avedro Mosaic CXL device (Avedro, Inc. Waltham, Massachusetts, United States).

#### Postoperative care

Postoperative topical medication is started at the day of surgery and is similar for both groups. Trafloxal (Ofloxacin (3 mg/ml), Bausch and Lomb) eye drops are prescribed 3 times daily for 1 week. Duratears (dextran 70 (1 mg/ml) and hyperomellose (3 mg/ml), Alcon) eye drops are prescribed 8 times daily. At the follow-up visit at day 4, the epithelial healing is evaluated. The bandage contact lens is removed if the epithelial defect is closed, and Trafloxal (Ofloxacin (3 mg/ml), Bausch and Lomb) is continued for another 3 days. One week after the CXL procedure FML Liquifilm (fluorometholon (1 mg/ml), Allergan) is started 2 times a day for 3 weeks. Duratears (dextran 70 (1 mg/ml) and hyperomellose (3 mg/ml), Alcon) is continued as needed.

#### Follow-up

A baseline measurement will take place during the preoperative visit for demographic variables, routine eye examinations, visual acuity, and corneal tomography. For both groups, these measurements will be repeated during postoperative visits. These follow-up study visits will be scheduled 4 days, 4 weeks, 3 months, 6 months, and 12 months after the crosslinking procedure. Additional visits will be planned if needed in case of complications. A more detailed overview of the study measurements per follow-up visit is presented in Table 1.

**Table 1:** Study procedures per follow-up time point

Assessment/Procedure	Preoperative visit	Follow-up visits				
		4 days	1 month	3 months	6 months	12 months
Check for in-/exclusion criteria	X					
Informed consent	X					
Medical history/ Demographics	X					
Check medication	X	X	X	X	X	X
Visual acuity	X		X	X	X	X
Refraction	X					X
Slit lamp examination	X	X	X	X	X	X
Fundoscopy	X					
IOP	X		X			
ECD	X				X	X
Pentacam	X		X	X	X	X
Anterior segment OCT			X			
Questionnaires						
• NEI VFQ-25	X			X	X	X
• KORQ	X			X	X	X
• EQ-5D-5L	X			X	X	X
• HUI-3	X			X	X	X
• Standardized cost questionnaire	X			X	X	X
• SF-MPQ		X				

*IOP* Intraocular pressure (mmHg), *ECD* Endothelial cell density (cells/mm^2^)

#### Outcome measures

The primary outcome is the mean change in Kmax 12 months after CXL procedure measured with the Pentacam® HR (OCULUS Optikgeraete GmbH, Wetzlar, Germany).

We also set multiple secondary objectives:

- Visual acuity: uncorrected and corrected distance visual acuity (UDVA and CDVA) in LogMAR (the Logarithm of the Minimum Angle of Resolution)
- Refraction
- Depth and size of demarcation line
- Pachymetry: change in thinnest corneal thickness
- Zonal Kmax: the analysis of a 3.0 mm zone centred on Kmax
- DUCK score: Dutch Crosslinking for Keratoconus Score is based on changes in 5 clinical parameters that are routinely assessed: age, visual acuity, refraction error, keratometry, and subjective patient experience. Each item is scored from 0 to 2 and cut-offs are determined by clinical experience [21].
- ABCD grading system: the Anterior radius of curvature (A), Posterior radius of curvature (B), Corneal pachymetry at thinnest point (C), Distance best corrected vision (D), and a modifier ( −) for no scarring, ( +) for scarring that does not obscure iris details and (+ +) for scarring that obscure iris details measured with the Pentacam HR [22].
- Success/failure rate: failure is defined as progression of the disease after CXL
- Mean endothelial cell loss
- Rate of reepithelialisation: evaluated at 4 days after CXL with fluorescein and blue light, a slit lamp image is taking to perform quantitative morphometric surface analysis
- Patient Reported Outcomes Measures (PROMs):
  - ∘ Vision specific quality of life and patient satisfaction will be measured using the National Eye Institute Visual Function Questionnaire-25 (NEI-VFQ-25) and the Keratoconus Outcome Research Questionnaire (KORQ)
  - ∘ Health-related quality of life (HRQL) will be measured using two questionnaires: EuroQol’s EQ-5D-5L and the Health Utilities Index Mark-3 (HUI-3)
  - ∘ Pain after CXL will be measured with the short form of the McGill Pain Questionnaire (SF-MPQ) during the first 4 days after CXL
- Incremental cost-effectiveness ratios (ICERs): These will be expressed as
  - ∘ incremental societal costs per quality-adjusted life year (QALY) gained based on the EQ-5D and HUI-3
  - ∘ incremental healthcare costs per patient with a reduction in Kmax of ≥ 1D after CXL, per clinically improved patient on the NEI VFQ-25 questionnaire, per clinically improved patient on the Keratoconus Outcome Research Questionnaire, and with clinical improvement in (un-) corrected distance visual acuity
- Budget impact: will be reported as the difference in costs resulting from implementation of cCXL, from the perspective of the budget holder. Different scenario’s will be compared to investigate the impact of various levels of implementation (i.e., 25%, 50%, 75% and 100% of eligible patients).

#### Adverse events

Adverse events are defined as any undesirable experience occurring to a subject ‘s eyes during the study, whether considered related to the trial procedure or the experimental intervention. All adverse events reported spontaneously by the subject or observed by the investigator, or his staff will be recorded, unless the event is of negligible impact and in addition has no connection to the study anywise.

The investigator will report all serious adverse events (SAE) to the sponsor without undue delay after obtaining knowledge of the events, except for the SAE’s unrelated to the intervention [23, 24]. The sponsor will report the SAEs through the web portal ToetsingOnline to the accredited medical ethical commission that approved the protocol, within 7 days of first knowledge for SAEs that result in death or are life threatening followed by a period of maximum of 8 days to complete the initial preliminary report. All other SAEs will be reported within a period of maximum 15 days after the sponsor has first knowledge of the serious adverse events.

### Statistical analysis

An electronic data capture program (Castor EDC) will be used to collect all data. Data analysis will be done with SPSS (SPSS Inc. Chicago, IL). Data analysis will be performed according to the intention-to-treat principle. Patients who reach a safety end point (e.g., additional intervention) and those lost to follow-up will still be included in the analysis. Per-protocol analysis will be performed as an addition to the intention-to-treat principle. Non-inferiority will be established if both analyses yield the same result.

Descriptive statistics will be used to present the baseline characteristics and a CONSORT diagram will describe the course of patients through the trial, details of the number of eligible patients, the number of informed consents and the number of randomized patients.

For the primary outcome, (the change from baseline in) Kmax, a linear mixed model analysis will be used, since it accounts for baseline differences, uses all available data, and corrects for correlation between repeated measures. Next to Group (cCXL vs. sCXL), Time and Interaction Group*Time, the stratification variable (Kmax) as well as variables related to the outcome and/or to missingness of outcome variable will be included in the fixed part of the model. For the secondary outcomes that are repeatedly measured, we will use the same model as for the primary outcome namely a linear mixed model to assess for overall differences between groups over time (quantitative, continuous data).

A trial-based economic evaluation will be performed within a time horizon of 12 months to estimate the cost-effectiveness (CEA) of cCXL compared to sCXL. Both a societal and healthcare perspective will be used. Incremental cost-effectiveness ratios (ICERs) will be calculated by dividing the difference in costs by the difference in effectiveness between cCXL and sCXL. A micro-costing method will be used to determine the costs in the study population. A detailed and complete analysis of the costs of each patient will be included in the study up to 12 months. The primary effectiveness measure used in the base case cost-effectiveness analysis is QALY. QALYs will be calculated based on generic HRQL, using two questionnaires: EuroQol’s EQ-5D-DL (base case analysis) and the HUI-3.

The budget impact analysis (BIA) will be performed to evaluate the impact of implementation of cCXL on the Dutch healthcare budget, compared to sCXL. The BIA will be performed in accordance with the Dutch guidelines for economic evaluations and the ISPOR guidelines (the Professional Society for Health Economics and Outcomes Research) [25, 26].

### Sample size calculation

For non-inferiority, we assume that the intervention group should have an average decrease in Kmax of 1.0 D, based on clinical experience and recent evidence. The non-inferiority margin is equal to -0.3 D (i.e., the mean difference between cCXL and sCXL should be greater than -0.3 to achieve statistical significance). Assuming this non-inferiority margin and a within-group standard deviation (SD) of 1.72 D, we need to include 55 patients per group to detect non-inferiority with 90% power and a one-sided significance level alpha of 0.025. The sample size was calculated using an online calculator for the comparison of two means (www.powerandsamplesize.com). Accounting for 10% loss-to-follow-up, we need to include 62 patients per group, i.e., 124 patients in total.

### Randomisation and blinding

Participants of the study will be randomized to either cCXL or sCXL (control), after applying the stratification factor of ‘Kmax < 58D’ or ‘Kmax ≥ 58D’. Each patient will receive a randomization number from a computerized random number generator. In Castor EDC, the subject number will be linked to one of the treatment arms (either cCXL or sCXL) through block randomization. Random varying block sizes of 2 and 4 will be used. The randomization procedure will be done by the principal investigator.

Since it is clear to both patients and surgeons which intervention will be performed, blinding will not be possible. The investigator that performs postoperative examinations will be blinded. Potential deblinding will be logged in the case report form (CRF).

### Data management and monitoring

Personal data will be managed confidentially, according to Good Clinical Practice guidelines. All essential documents, including informed consent forms, CRF, and questionnaires will be collected by the principal investigator of each study centre and will be stored in an investigator site file for a period of 15 years. CRFs will be documented electronically in Castor, by a member of the study team of each study centre. The Clinical Trial Centre Maastricht (CTCM) will monitor the study to protect patient rights and accuracy of reported trial data. The CTCM is an academic research organisation.

### Public disclosure and publication policy

The results of this study will be published in report form and scientific papers will be submitted for publication in appropriate journals in the international medical literature. The guidelines for public disclosure of WMO-studies, as stated by the Central Committee on Research Involving Human Subjects (CCMO) in their publication statement, will be followed. The results of this RCT will be reported according to the CONSORT (CONsolidated Standards Of Reporting Trials) statement.

### Data safety monitoring board

In this study a Data Safety Monitoring Board (DSMB) is established to perform ongoing safety surveillance and to perform interim analysis on the safety data, especially regarding the occurrence of an SAE. The DSMB is an independent committee of trial experts who will focus on safety monitoring, it consists of three members: two ophthalmologists and one member with a statistical/epidemiological background. The DSMB decides whether to recommend that the trial continues to recruit participants or whether recruitment should be terminated either for everyone or for some treatment groups and/or some participant subgroups. The DSMB may perform an interim analysis on safety, upon the suspicion of one of the treatments being more harmful than the other. Interim analyses on safety will be performed at least three times yearly. The advice(s) of the DSMB will only be sent to the coordinating investigators group of the study. Should the coordinating investigators group decide not to fully implement the advice of the DSMB, they will send the advice to the reviewing Medical Ethical Commission, including a note to substantiate why (part of) the advice of the DSMB will not be followed.

## Discussion

At the moment, corneal crosslinking is the only treatment to halt progression in keratoconus. Recent ultra-structural research has shown that keratoconus affects the cornea locally and does not affect the entire cornea. Seiler et al. used a customized treatment pattern with energy levels ranging from 5.4 J/cm^2^ to 10 J/cm^2^centred on the maximum posterior elevation. They found flattening and regularization of the cornea at 12 months and a faster epithelial healing period [19]. Nordström et al., who used a customized treatment pattern with energy levels ranging from 7.2 J/cm^2^ to 15 J/cm^2^centred on the maximum corneal steepness, found improved corneal flattening and improved visual acuity compared to a uniform CXL treatment [18]. Cassagne et al., using topography-guided crosslinking with energy levels ranging from 5.4 J/cm^2^ to 15 J/cm^2^, found a faster healing time and significant improvement at 12 months both in maximum and mean keratometry, and in best corrected visual acuity [17]. Shetty et al. investigated three different customized treatment patterns and showed greatest cornea flattening and improvement in uncorrected and corrected visual acuity with a customized pattern based on tangential curvature maps. Thus, based on these studies, only treating the affected zone appears to have three potential benefits: (1) a faster recovery (e.g., increased corneal reepithelialisation), (2) stronger flattening of Kmax and (3) better visual outcome (corrected distance visual acuity, CDVA). However, these studies were small and non-randomized.

We will set up a randomized controlled trial to evaluate if customized CXL is non-inferior to standard CXL in terms of corneal flattening and halting keratoconus progression. For our customized treatment pattern, we will develop our own program to determine cone location using data from the Pentacam Scheimpflug HR device. If necessary, it would be possible to adjust our program to other tomography devices. To perform the customized treatments, we will use the Avedro Mosaic CXL device (Avedro, Inc. Waltham, Massachusetts, United States). This could be a possible obstacle for implementation since not every CXL device is equipped with a feature to customize treatments. Additionally, we will evaluate if cCXL offers additional benefits on quality of life through different questionnaires and we will perform a cost-effectiveness analysis to calculate and compare the costs of both procedures.

## Data Availability

The datasets used and/or analysed during the current study are available from the corresponding author (MV) on reasonable request.

## Abbreviations

AE: Adverse event
BIA: Budget impact analysis
cCXL: Customized crosslinking
CDVA: Corrected distance visual acuity
CEA: Cost-effectiveness analysis
CRF: Case report form
CTCM: Clinical Trial Centre Maastricht
CXL: Crosslinking
D: Dioptre
DSMB: Data Safety Monitoring Board
HUI-3: Health Utilities Index Mark-3
HRQL: Health-related quality of life
ICER: Incremental cost-effectiveness ratios
K1: Flat keratometry
K2: Steep keratometry
Kmax: Maximum curvature
KORQ: Keratoconus Outcome Research Questionnaire
LogMAR: Logarithm of the Minimum Angle of Resolution
NEI-VFQ-25: National Eye Institute Visual Function Questionnaire-25
PROMs: Patient Reported Outcomes Measures
QALY: Quality-adjusted life years
SAE: Serious adverse event
sCXL: Standard crosslinking
SD: Standard deviation
SF-MPQ: Short form of the McGill Pain Questionnaire
UDVA: Uncorrected distance visual acuity
WMO: Medical Research Involving Human Subjects Act
